# Improvement of persistent impairments in executive functions and attention following electroconvulsive therapy in a case control longitudinal follow up study

**DOI:** 10.1186/s12888-024-06270-5

**Published:** 2024-11-20

**Authors:** Åsa Hammar, Eivind Haga Ronold, Malene Alden Spurkeland, Rita Ueland, Ute Kessler, Ketil J. Oedegaard, Leif Oltedal

**Affiliations:** 1 Department of Biological and Medical Psychology, University of Bergen, Alrek helseklynge Årstadveien 17, Bergen, 5020 Norway; 2 Department of Clinical Sciences Lund, Psychiatry, Faculty of Medicine, Lund University, Lund, Sweden; 3 Office for Psychiatry and Habilitation, Psychiatry Research Skåne, Region Skåne, Sweden; 4 Department of Clinical Medicine, University of Bergen, Bergen, Norway; 5 Division of Psychiatry, Haukeland University Hospital, Bergen, Norway; 6 Mohn Medical Imaging and Visualization Centre, Department of Radiology, Haukeland University Hospital, Bergen, Norway

**Keywords:** ECT, Short- long-term effects, Longitudinal, Iatrogenic effects, Executive function, Processing speed, Attention, Treatment-resistant depression, Major depressive disorder

## Abstract

**Background:**

How cognition is influenced by electroconvulsive treatment (ECT) and major depressive disorder (MDD) is still debated. The development and etiology of neurocognitive impairment in MDD were examined by investigating the cognitive profile following ECT related to the state, scar, and trait perspectives, with the former predicting improvements parallel with depressive symptoms, while the two latter expected persisting impairments. Executive functions (EF) and attention are central to cognition and alterations in these functions could influence other domains like memory. The main aims of the present study were to examine the short and long-term effects of ECT on EF and attention in patients with major depressive disorder by exploiting the rapid antidepressant effect of this treatment.

**Methods:**

A case-control longitudinal follow-up design was used to investigate the effects of unilateral brief-pulse ECT on EF and attention in patients with depression (*n* = 36) compared to untreated healthy controls (*n* = 16). EF and attention were measured pre-treatment, approximately two weeks, and six months post-treatment.

**Results:**

The patient group showed significantly worse performance on most tests compared to healthy controls pre-treatment, and no short- or long-term worsening of EF and attention following ECT was found. Significant improvement was identified in patients’ attention, processing speed and inhibition after ECT.

**Conclusions:**

The present study showed that there was no cognitive worsening after ECT treatment. An improvement in several of the tests measuring inhibition, attention, and processing speed was parallel to symptom reduction, with the former showing associations to symptom change, suggesting state-related effects from improved mood. Still, the patient group performed significantly worse on most measures both pre-treatment and at the short and long-term follow-ups, indicating prevailing trait or scar effects on cognitive functions and potential lack of practice effects.

**Clinical trial number:**

NCT04348825 (14.04.20).

## Background

Depression is the most prevalent mental disorder, and studies indicate that between 10 and 30% of the population will be diagnosed with Major Depressive Disorder (MDD) during a lifetime [1, 2]. The disorder is one of the leading causes of disability worldwide and largely contributes to the global burden of disease [3], through substantial functional impairment [4, 5]. Although there are effective treatments targeting depression, over one-third of patients do not respond to first-line interventions, such as anti-depressant medications or psychological interventions, and could be considered treatment-resistant [6]. Electroconvulsive therapy (ECT) represents one of the most effective treatments for severe depression [7–10], with high remission rates even in cases of treatment-resistant depression [3, 6, 11]. However, ECT is often used as a treatment of last resort [12, 13], possibly due to stigma [14, 15], and concerns about adverse side effects [16]. Despite accumulating evidence of its safety and effectiveness, it is still unclear to what degree ECT may cause persistent neurocognitive deficits in some patients treated for depression [15]. Enhanced knowledge about possible short- and long-term side effects of ECT is considered crucial for improving clinicians’ and patients’ ability to make informed decisions about treatment.

### Cognitive deficits in major depressive disorder and their course

MDD is associated with cognitive impairments [15–18]. Deficits persist in remission and worsen with the duration of depressive episodes [17, 18]. Executive functions (EF) and attention are two of the neurocognitive domains significantly affected in patients suffering from depression [19–21], with impairments still evident in remission [18, 22]. EF are needed to manage most of our daily activities and underlie our ability to organize thinking [23, 24], self-regulate, plan, inhibit and make decisions [25]. When EF is measured with standardized neuropsychological tests, people with MDD tend to perform significantly worse compared to healthy controls on tasks measuring aspects of EF such as shifting, inhibition, and verbal fluency in the acute state of depression [21, 25–27], with lower performance persisting beyond the depressive episode [18, 22, 26, 28, 29].

Similar to EF, attention is an associated multidimensional construct and a selection mechanism [30], that allows *control* of limited cognitive resources in a flexible manner [31]. Attention is closely related to how fast information is processed and responded to, which is commonly termed processing speed [32]. There is support in the literature for attentional deficits during the acute state of depression [27], although findings are inconsistent regarding which aspects of the attentional domain that are affected [33–35]. In addition, there are findings indicating that the severity of depressive symptoms could influence the extent of impairment [33]. Although attentional deficits are shown to persist in remission [18, 36], they are smaller compared to the acute state [15, 34, 35, 37], and recent studies suggest that attentional functioning improves following treatment [17, 37, 38], indicating a possible state-effect of depressive symptoms on attention. However, various aspects of attention could be influenced differently, and patient group characteristics, such as severity of depression and duration are related to both improvement and impairment [17, 35, 39–41]. Investigating aspects of attention pre- post ECT could provide insight into the relationship between attention and depressive symptoms and severity, as well as the impact of ECT.

Deficits could be either a pre-existing vulnerability (trait), relate to the depressive symptom load (state), or a consequence of progressive scarring due to the neurotoxic effects of depression (scar) [23, 36]. There is some support in the literature for all three perspectives [36], however, the literature is mainly based on studies following patients treated with antidepressants and/or psychological treatments [17], and less is known about the effects on populations that do not respond to such treatments which could necessitate ECT Thus, examining changes in different domains of cognition following the rapid ECT-treatment effect of depression is warranted and could inform on these perspectives.

### Electroconvulsive therapy and cognition

Research on depressed patients treated with ECT does not indicate lasting treatment-induced deficits in objective measures of EF and attention [11, 42–46]. However, an acute decline in attention and the executive aspect of inhibition was observed at the initiation of ECT, but normalized after completion of treatment sessions [47]. Small decreases have been reported in verbal fluency shortly after ECT in heterogeneous patient samples [15]. Small to medium improvements have been reported for shifting, inhibition and planning, and recovery of baseline levels for verbal fluency in long-term measures post ECT [15, 42]. Attentional functioning is shown to improve both short- and long-term following ECT [15, 44, 45], particularly in the aspects of processing speed and working memory [9, 44, 45, 47]. This could indicate state effects and is similar to improvements seen in MDD populations receiving other treatments [17]. However, executive and attentional functioning in patients is still not comparable to healthy controls post-ECT [15, 47], making it difficult to conclude regarding the effects of ECT versus the effects of depression in relation to the state, trait, and scar hypotheses.

Importantly, there are several methodological shortcomings concerning previous research examining the effects of ECT on cognition that constrain the state of knowledge [15, 48]. Firstly, there are few studies investigating the longitudinal effects of ECT on cognition [15, 45, 48]. Previous studies also lack the inclusion of a healthy control group, which makes controlling for practice effects due to repeated testing challenging [11, 47]. Studies also vary regarding how ECT as an intervention is applied, the timing of measurements and neuropsychological assessments [15]. In addition, both EF and attention could influence other cognitive functions like memory [35, 49], and thus the development of these functions following treatment is important for separating the cognitive residual symptoms related to depression history from side effects reported following ECT. Lastly, results from the same neuropsychological tests are being reported and interpreted differently, partly due to assumptions that the same tests are tapping multiple cognitive processes [25, 32]. In the present study, these issues are reduced by including several outcome measures from both the executive and attentional domains.

Prior research has faced challenges in effectively differentiating the effects of depression and ECT on cognitive functioning. This is rectified in the present study by including baseline measures and a healthy control group, to examine whether side effects identified post-ECT are treatment-induced, or if findings coincide with research demonstrating long-term cognitive impairment following severe depression [18, 26, 28, 29, 36, 48]. In addition, by utilizing the rapid antidepressant effect of ECT [9, 48], our design allows for innovative exploration of the scar, trait, and state perspectives in relation to the domains of executive and attentional functioning. While the state perspective would predict recovery of cognitive functions following ECT and symptom relief, the trait and scar perspectives would predict preexisting, lasting neurocognitive deficits, and even exacerbation of cognitive functions. Including a variety of measures and separating the domains of EF and attention enables the identification of specific cognitive profiles in patients undergoing ECT.

## Aims, expectations and research questions

The main aim of this study is to investigate short- and long-term effects of ECT on both executive and attentional functioning in patients with major depression related to the state, scar, and trait perspectives. We expect patients with MDD to have significantly poorer performance on all tests measuring attention and EF at baseline compared to healthy controls. Patients’ performances are expected to either improve or remain stable shortly post-treatment. At six-month follow-up, we expect long-term improvement in the aspects of processing speed and working memory. However, we still expect patients to perform significantly worse compared to healthy controls. It is therefore expected that we will find both state-related function improvements, but also lasting impairment that is in line with trait- or scar-related effects of depression. The following research questions were formulated:

1. Do patients suffering from depression have reduced executive functions, processing speed and attention when compared to healthy controls pre ECT?
2. Do patients´ executive processing speed and attentional functions improve shortly and long-termly (6 months) post-ECT?
3. Are patients’ executive processing and attentional abilities comparable to healthy controls shortly post ECT and at 6 months follow-up?
4. Will the cognitive profile following ECT show improvement in EF processing speed and attention related to mood symptoms (state) or show pre-post persistent impairments (trait or scar)?

## Methods

### Study design

A pre-post intervention longitudinal follow-up study was conducted [50], and data were collected at baseline approximately one week before receiving ECT (T1), 2 weeks post-treatment (T2), and 6 months post-treatment (T3). See Table 1 for the mean follow-up time in days. Patients were compared to a healthy control group that underwent repeated neuropsychological testing at the same time points as the patients. The study was approved by the Regional Committee for Medical and Health Research Ethics, REC South East, Norway (2013/1032) and conducted in accordance with the Helsinki Declaration. All participants gave their signed informed consent before participating in the study. Clinical trial number: NCT04348825 (14.04.20).

### Participants and procedure

The original patient group consisted of forty (*n* = 40) participants who were referred to Haukeland University Hospital, Bergen, Norway, for ECT. Eligibility criteria included patients over the age of 18, with no upper age limit for participation, referred to ECT due to a moderate or severe depressive episode according to guidelines from the Norwegian Health Department [51]. These guidelines recommend referral for patients with moderate to severe depression who have shown a lack of effect from conventional treatments, with psychotic symptoms, and/or experience life-threatening depression due to suicidality. All patients fulfilled the criteria for one of the following ICD-10 diagnoses according to interviews with referring clinicians (psychologists/psychiatrists) in specialist mental health services: F31.3 Bipolar disorder, current episode depressed, moderate severity (10,3%) and F31.4 Bipolar disorder, current episode depressed, severe, without psychotic features (7,69%); F32.1 Major depressive disorder, single episode, moderate (2,56%) and F32.3 Major depressive disorder, single episode, severe with psychotic features (5,13%); F33.1 Major depressive disorder, single episode, severe with psychotic features (12,32%) and F33.2 Major depressive disorder, recurrent, severe without psychotic features (56,41%) and F33.3 Major depressive disorder, recurrent, severe with psychotic symptoms (5,13%). In addition, Patients had to score 25 or higher on the Montgomery Aasberg Depression Rating Scale (MADRS). Patients with both bipolar and unipolar depression were included. Exclusion criteria were pregnancy, an intelligence quotient (IQ) of ≤ 70, and a history of receiving ECT within the last 12 months. One patient was excluded due to an IQ score under 70. Twenty-six (*n* = 26) patients underwent neuropsychological testing at T1. Twenty (*n* = 20) patients returned for testing at short-term follow-up (T2), with a late inclusion of ten (*n* = 10) patients, resulting in a total of thirty patients measured at T2 (*n* = 30). A total of nineteen (*n* = 19) patients returned for 6-month follow-up (T3).

The control group consisted of sixteen (*n* = 16) healthy controls of equivalent mean age and sex distribution as patients. Fifteen (*n* = 15) participants from the control group were assessed with the neuropsychological testing at T1, sixteen (*n* = 16) were assessed at T2, and fifteen (*n* = 15) participants returned for 6 months follow-up. Details on participant demographics are listed in Table 1.

**Table 1:** Demographics at baseline for all participants and attrition for patients

Variable	Patients (*n* = 36)		Controls (*n* = 16)		Attrition Patients	
	Mean	SD	Mean	SD	Mean T2 (*n* = 5)	Mean T3 (*n* = 12)
Age	45.06	14.21	42.06	16.23	42.2	40.42
Male/female	18/18	NA	5/10	NA	NA	NA
Education	13.75*	3.32	16.31*	2.22	10.8**	13.5
Total IQ	106.22*	13.09	120*	7.17	97.6	110
MADRS T1	33.54*	5.26	1.92*	2.29	30.2	34.33
MADRS T2	15.08*	8.88	1.08*	1.44		
MADRS T3	14.5*	11.06	0.77*	1.42		
T1 MMSE	28.46	1.96				
T1 NP	4.31	1.89				
T2 NP	16.83	11.22				
T3 NP	196.7	61.84				

MADRS, Montgomery Aasberg Depression Rating Scale; NA, not applicable. MMSE, Mini-mental state examination. NP, days between neuropsychological assessment and ECT *Means that are significantly different between groups with (*p* < .01)

Attrition: ** Means on variables where drop-out participants are significantly different (*p* < .05)

### ECT treatment

Patients received three sessions with ECT per week until remission, or until the maximum of 18 sessions was reached. Patients underwent right unilateral electrode placements with a Thymatron System IV (Somatics LLC, Lake Bluff, Illinois) providing brief-pulse, square wave, constant current. The initial stimulus energy was calculated based on gender and age, and the duration of the stimulus pulse was set to 0.5 ms. Thiopental or Propofol was given as anesthesia. Patients were hyperoxygenated with oxygen-enriched air, minutes before and during anesthesia to optimize the induction of seizures. The adequacy of each seizure was evaluated by an ECT clinician based on duration, reorientation time, clinical effect, and d-waves. Seizures that were evaluated by the ECT clinician as not effective led to restimulation in the same session, and/or adjustment of the stimulus parameter in the following session. For specific information about the ECT treatment, see [50].

## Clinical measures

The severity of the current depressive episode was verified and monitored by the MADRS scale [52] at T1, T2 and T3 (See Table 1).

### Neurocognitive assessment

Neuropsychological tests were administered by a trained technician and consisted of standardized tests measuring attentional resources and executive functions. Wechsler Abbreviated Scales of Intelligence [53] was administered as an estimate of general abilities (IQ).

#### Executive functioning

Four tests from the Delis-Kaplan Executive Function System (D-KEFS [54]) were administered to measure aspects of executive functioning, with seven outcome variables included in the analysis. In addition, the Wisconsin Card Sorting Test (WCST [55]) and the Continuous Performance Test-II (CPT [56]) were included.

**Inhibition** was measured using the Color-Word Interference Test (CWIT). In the third condition of CWIT, measuring inhibition, participants are expected to identify the color ink of the word and suppress the response to read the word. Commissions on CPT were used as a measure of inhibition ability. The number of commission errors is the number of times participants incorrectly press the key when seeing “X” and is one of several outcomes from the CPT [56]. High scores correspond to poor performance in both the inhibition tasks.

**Mental Flexibility or Shifting** was measured using the fourth condition of CWIT, inhibition/switching, where participants are asked to switch from color naming of an incongruent word (CWIT 3) to reading (CWIT 2) when the word appears within a frame. In this task, high scores correspond to poor performance. In addition, the Category Switching condition of the Verbal Fluency test was included to measure flexibility and shifting. Category switching measures participants’ ability to alternate between naming members of different categories, where higher scores equal better performance. Shifting was also measured using the number-letter switching condition of the D-KEFS Trail Making Test Part 4 (similar to TMT-B). In TMT- 4 participants have to shift between connecting letters and numbers, measured as time to complete the task. Therefore, higher scores correspond to poorer performance. In the WCST participants are asked to sort cards by different dimensions, such as color or shape, which demands maintaining or switching of rules in response to feedback. The number of successful switches between category sets and perseverative errors, which is applying an older previous rule for sorting, was used as an estimate for shifting ability. A high score on the WCST perseverative errors would indicate poor performance, while the contrary is true for the WCST categories completed.

**Verbal Fluency** was measured using the D-KEFS Verbal Fluency Test consisting of the following conditions: Letter Fluency and Category Fluency. Letter fluency is measured by asking patients to generate as many words as possible starting with a certain letter, while semantic verbal fluency is naming as many words as possible from a semantic category. In both measures of verbal fluency, higher scores equal good performance.

**Planning ability** was measured using the Total Achievement Score of the Tower Test. Using as few moves as possible, the task of the D-KEFS Tower Test is to reproduce a pictured tower model, following a set of rules. For the Tower Test, higher scores indicate greater performance.

#### Attentional functioning and processing speed

Four tests were administered to measure aspects of attentional resources; Digit Symbol Substitution Test (DSST [53]) Digit Span Test (DST [53]), omission errors in CPT, and failure to maintain set in WCST.

**Auditory Attention and Working Memory** were measured with DST. A sequence of numbers is read aloud to participants, who are then asked to repeat the sequence in the same (attention) or backward (working memory) order as presented. The number sequences get progressively longer. Low scores on DST equals poor performance.

**Visual Attention and Processing Speed** were measured with the DSST. Participants are asked to match symbols to numbers according to a template. Within a given time frame, participants copy the symbols into empty spaces below a row of numbers. The number of correct symbols within the time frame constitutes the score, where low scores equal poor performance.

**Inattention** was measured with omission errors in CPT and failure to maintain set in WCST. In CPT participants are asked to press a key every time a letter appears on a computer screen, except for the letter “X”. Failure to press the key when a letter is displayed is an omission error and indicates inattentiveness or a lack of sustained attention. Failure to maintain set in the WCST is measured by the number of times a participant incorrectly changes their sorting strategy after the rule is known and before the change is appropriate. High scores equal poor performance in both inattention tasks.

### Statistics, contrast and change scores

All statistical analyses were performed in IBM SPSS Statistics version 28 (IBM Corp, Armonk, New York). One-way ANOVA tests were performed to assess differences between groups in demographic variables, symptomatology, results on neuropsychological tests at all time points, attrition effects from the patients that dropped out, and differences between change scores. A one-way between-group ANOVA was conducted to explore the impact of ECT on EF and attention in patients with MDD. Outcome measures were assessed for normality and Mann-Whitney U-tests were used as a nonparametric alternative to examine group differences in performance on three outcome variables of the WCST (preservative errors, failure to maintain set, categories completed) and on CPT omissions, and Lenhard et al. [57] was used to calculate eta squared for these tests. All variables consisted of raw scores, except for CPT omissions/commissions where t-scores were used. Paired-samples t-tests were conducted to evaluate the impact of ECT on EF and attentional functioning in patients with major depression. Paired-samples t-tests were also conducted on means for the control group to detect retest/practice effects. Results are not corrected for multiple comparisons, as conducting a Bonferroni correction would result in low power to detect significant findings due to the small sample size and drop-out rates in the study. Contrast scores for investigating EF relatively independent from processing speed [29], were calculated by the following formulas: Inhibition contrast = CWIT 3 – (CWIT 1 + CWIT 2)/2, Mental/flexibility contrast = CWIT 4 – (CWIT 1 + CWIT 2 + CWIT 3)/3, Shifting contrast = TMT 4 – (TMT 1 + TMT 2 + TMT 3 + TMT 5)/4. Change scores were calculated by subtracting follow-up test results and MADRS scores from baseline so that positive values indicated improvements [58]. Bivariate correlation coefficients were calculated for change scores in neuropsychological tests and depressive symptoms. The significance level was set at *p* = .05. Effect sizes were described as small, medium, and large according to Cohen [59].

## Results

### Demographic variables, clinical measures and attrition effects

One-way ANOVA revealed that the patient group had a significantly lower IQ and fewer years of education in comparison with the control group (See Table 1). Regarding attrition effects, patients lost to follow-up from T1 to T2 had significantly fewer years of education compared with participating patients. There were no significant differences in any of the other demographic variables for patients lost to follow-up.

### Cognitive deficits pre electroconvulsive therapy

There were significant differences between the groups in performance on the EF tests CWIT-3, TMT-4, Category Fluency, and Tower, with medium to large effect sizes (See Table 2 for means and statistics), and differences remained significant when controlling for processing speed (EF contrast scores). Differences in CWIT-4, letter fluency, and WCST perseverative errors approached significance with medium effect sizes. There were also significant differences between groups on the attention and processing speed tests DST, DSST, and CPT omissions at T1. Effect sizes on these tests were medium to large. In sum, patients performed worse than controls on all measures of EF pre-ECT, and the differences were significant on all variables except for the CWIT-4 contrast score, category switching, CPT commissions and WCST categories completed, and WCST failure to maintain set.

**Table 2:** Differences between groups at T1, T2 and T3

Time	Pre-ECT (T1)					Post-ECT (T2)					6-months (T3)				
Groups *n* (male/female)	Patients *n* = 26 (18/18)	Control *n* = 15 (5/10)	Statistics			Patients *n* = 30	Control *n* = 16	Statistics			Patients *n* = 19	Control *n* = 15	Statistics		
Outcome variable	M(SD) /Mr	M(SD) /Mr	F(df) /U	*p*	Eta sq.	M(SD) /Mr	M(SD) /Mr	F(1) /U	*p*	Eta sq.	M(SD) /Mr	M(SD) /Mr	F(1) /U	*p*	Eta sq.
**EF**															
CWIT-3 Inhibition	70* (28.4)	49.8* (9.1)	**7.1 (1**,**38)**	**0.011**	**0.157**	67.8* (27.5)	47.8* (9.1)	**8.289 (1**,** 44)**	**0.006**	**0.159**	66.3* (29.7)	49* **(9.7)**	**4.672 (1**,**32)**	**0.038**	**0.127**
CWIT-3 Contrast scores	38.9* (23.1)	23.4* (6.1)	**6.5**	**0.015**	**0.146**	36.6* (22.7)	21.4* (6.2)	**6.811**	**0.012**	**0.134**	36.42 (25.81)	23.27 (8.44)	3.572	0.068	0.100
CWIT-4 Inhibition/switching	73.2 (28.3)	58.7(13.1)	3.501	0.069	0.084	78.8* (32)	55.2* (11.7)	**8.07**	**0.007**	**0.155**	70.11* (30.75)	51.93* (10.08)	**4.802**	**0.036**	**0.13**
CWIT4 Contrast scores	29.2 (17.5)	24.4 (9.2)	0.947	0.337	0.024	35.3* (22.9)	22.1* (8)	**5.002**	**0.03**	**0.102**	28.07 (20.9)	18.44 (8.29)	2.817	0.103	0.081
Letter Fluency	39.9 (11.1)	46.4 (12.1)	3.026	0.090	0.074	36.6* (12.3)	49.6* (13.3)	**10.764**	**0.002**	**0.2**	33.9* (11.21)	53.47* (13.47)	**21.391**	**< 0.001**	**0.401**
Category Fluency	41* (8.4)	52.7* (10.1)	**15.663**	**< 0.001**	**0.292**	40.2* (9.5)	52.2* (9)	**17.020**	**< 0.001**	**0.284**	38.11* (8.01)	51.4* (9.16)	**20.353**	**< 0.001**	**0.389**
Category Switching	13.8 (2.7)	14.7 (3.4)	0.784	0.382	0.020	12.55* (2.76)	14.4* (2.6)	**4.68**	**0.036**	**0.098**	12.16* (2.36)	15.53* (2.47)	**16.408**	**< 0.001**	**0.339**
TMT-4	100.3* (42.5)	61.6* (19.1)	**10.978**	**0.002**	**0.229**	100.8* (47)	63.1* (27.4)	**8.606**	**0.005**	**0.167**	100.22* (46.06)	65.87* (26.04)	**6.57**	**0.015**	**0.175**
TMT-4 Contrast scores	76.7* (37.7)	44.3* (17.9)	**9.641**	**0.004**	**0.207**	77.2* (40.2)	45.9* (23.5)	**8.141**	**0.007**	**0.159**	76.07* (38.19)	49.11* (21.78)	**5.866**	**0.021**	**0.159**
Tower Total Achievement	16.9* (3.3)	20.1* (3.7)	**8.226**	**0.007**	**0.178**	17.6* (3.3)	20.8* (3.1)	**9.482**	**0.004**	**0.184**	17.78* (4.32)	22.8* (4.16)	**11.434**	**0.002**	**0.269**
CPT Commissions (t-scores)	55.9 (13.4)	50.2 (10)	1.981	0.167	0.05	49.6 (11.1)	44.5 (7.5)	2.744	0.105	0.06	51.48* (9.54)	43.41* (6.21)	**7.801**	**0.009**	**0.206**
WCST Categories Completed	19.96	21.4	201	0.72	0.004	22.69	23.56	241	0.666	0.001	16.19	17.97	149.5	0.606	0.008
WCST Perseverative Errors	23	16.33	125	0.083	0.076	**26.72**	**16.25**	**124**	**0.01**	**0.146**	**20.92**	**12.3**	**64.5**	**0.009**	**0.197**
**Attention**															
DST	12.04* **(2.14)**	13.93* **(2.93)**	**5.64**	**0.022**	**0.126**	12.65 (2.56)	13.37 (2.68)	0.78	0.380	0.018	12.84 (2.65)	14.06 (3.86)	1.19	0.282	0.036
DSST	43.31* **(14.38)**	**59**,93* **(11.68)**	**14.47**	**< 0.001**	**0.271**	44.58* **(17.68)**	63.62* **(14.64)**	**13.42**	**< 0.001**	**0.238**	46.21* **(14.69)**	65.20* **(12.20)**	**16.19**	**< 0.001**	**0.336**
CPT omission (t-scores)	**23.86**	**14.90**	**103.5**	**0.018**	**0.138**	25.74	18.03	152.5	0.059	0.079	**19.56**	**13.03**	**75.5**	**0.049**	**0.12**
WCST Failure to Maintain Set	21.52	18.80	162.0	0.489	0.013	22.24	24.38	254.9	0.563	0.006	18.11	15.67	115.0	0.486	0.016

*Explanations*: CPT Commissions, t-scores; Tower, Tower Total Achievement Score

*Abbreviations*: Mr = Mean Rank

*Means that represent a statistical significant difference with *p* < .05

### Cognitive deficits following electroconvulsive therapy

At T2, there were statistically significant differences between groups in the following measures of EF CWIT-3, CWIT-4, Letter Fluency, Category Fluency, Category Switching, TMT-4, Tower and WCST preservative errors (See Table 2 for means and statistics). The effect sizes were medium to large. At T3 differences were still statistically significant between groups on these tests, in addition to CPT Commissions, with tests showing medium to large effect sizes. In addition, groups were significantly different on DSST with large effect sizes at both T2 and T3 Also, A medium significant difference between groups in performance on CPT omissions, at T3. In sum, there were significant short- and long-term differences between groups in all EF measures except WCST categories completed, and short-term and long-term significant differences between groups in processing speed, in addition to long-term differences between groups in inattention.

### Changes in cognition following electroconvulsive therapy

#### Short-term change

Patients demonstrated a statistically significant improvement in performance on CWIT-3 from T1 to T2 with a medium to large effect (See Table 3 for means and statistics). Controls also showed significant improvement in CWIT-3 from T1 to T2 with a medium to large effect. Also, contrast scores for CWIT-3 showed significant improvement for the patient group with a medium to large effect size. Patients demonstrated a medium improvement on the Tower test. A possible decline in verbal fluency switching appeared in the patient group, which approached significance. Both groups showed significant improvement in CPT Commissions from T1 to T2. There were no significant changes in attentional performance in the patient group from T1 to T2. The control group showed a statistically significant increase in performance on DSST from T1 to T2 with a large effect. In sum, the patient group and the control group showed significant short-term improvement in Inhibition indicating practice effects in both groups. Only the control group showed significant short-term improvement in some of the other functions.

#### Long-term change

Patients demonstrated a medium- and controls a large statistically significant improvement in performance on CWIT-4 from T2 to T3. Patients showed a statistically significant improvement in CWIT-3 and CWIT-3 contrast scores from T1 to T3 with large effect sizes. Patients also approached significant change in CWIT-4 with a large to medium effect size. Patients showed a statistically significant increase in performance on DST and DSST from T2 to T3 with a medium to large effect size. Controls showed a statistically significant improvement in CPT omissions with a moderate effect size. From T1 to T3 patients showed a statistically significant increase in DSST with a large effect size. The patient group showed a significant large increase in WCST failure to maintain set.

**Table 3:** Mean change between time points in both groups. Paired samples t-test

Time	T1 - T2								T2 - T3								T1 - T3							
	Patient group *n* = 20				Control group *n* = 15				Patient group *n* = 17				Control group *n* = 15				Patient group *n* = 12				Control group *n* = 14			
	MC	t (df)	*p*	*d*	MC	t (df)	*p*	d	MC	t (df)	*p*	d	MC	t (df)	*p*	d	MC	t (df)	*p*	d	MC	t (df)	*p*	d
**EF**																								
CWIT-3 Inhibition	**7.53**	**2.878 (18)**	**0.01**	**0.66**	**3.07**	**2.57 (14)**	**0.022**	**0.664**	2.06	0.67 (17)	0.512	0.158	− 0.73	− 0.472 (14)	0.644	− 0.122	**11.55**	**2.445 (10)**	**0.035**	**0.737**	2.21	1.179 (13)	0.26	0.315
CWIT-3 Contrast scores	**6.71**	**3.297 (18)**	**0.004**	**0.756**	2.4	1.948 (14)	0.072	0.503	− 0.11	− 0.037 (17)	0.971	− 0.009	-1.2	− 0.697 (14)	0.497	− 0.18	**8.68**	**2.646 (10)**	**0.024**	**0.798**	0.89	0.491 (13)	0.632	0.131
CWIT-4 Inhibition/ switching	-1.1	− 0.288 (18)	0.777	− 0.066	3.8	1.93 (14)	0.074	0.498	**12.67**	**3.053 (17)**	**0.007**	**0.72**	**4.07**	**3.201 (14)**	**0.006**	**0.827**	11	2.170 (10)	0.055	0.654	**7.79**	**3.166 (13)**	**0.007**	**0.846**
CWIT-4 Contrast scores	-4.16	-1.275 (18)	0.218	− 0.293	2.33	1.035 (14)	0.318	0.267	10.54	2.734 (17)	0.014	0.644	**4**	**3.376 (14)**	**0.005**	**0.872**	5.24	1.383 (10)	0.197	0.417	**6.17**	**2.339(13)**	**0.036**	**0.625**
Letter Fluency	0.47	− 0.214(18)	0.833	− 0.049	**4.13**	**-2.248 (14)**	**0.041**	**− 0.581**	0.71	− 0.25 (16)	0.806	− 0.061	3.2	-1.962 (14)	0.07	− 0.507	-1.73	0.407 (10)	0.693	0.123	**8.36**	**4.216(13)**	**0.001**	**-1.127**
Category Fluency	− 0.68	0.507 (18)	0.618	0.116	0.00	0.00 (14)	1.00	0.00	1.47	-1.003 (16)	0.331	− 0.243	− 0.93	0.561 (14)	0.584	− 0.145	− 0.73	0.288	0.779	0.087	− 0.64	0.289 (13)	0.777	0.077
Category Switching	-1.21	2.051(18)	0.055	0.47	0.133	0.195 (14)	0.848	0.05	− 0.0588	0.142 (16)	0.889	0.034	1.13	-1.476 (14)	0.162	− 0.381	-1.27	1.249(10)	0.24	0.377	1.07	-1.069 (13)	0.305	− 0.286
TMT-4	7.94	1.54 (17)	0.142	0.363	1.2667	0.281 (14)	0.78	0.073	5.82	0.786 (16)	0.443	0.191	− 0.47	− 0.174 (14)	0.865	− 0.045	7.27	0.929 (10)	0.375	0.28	0.71	0.135 (13)	0.895	0.036
TMT-4 Contrast scores	5.88	1.208 (17)	0.244	0.285	1.01	0.219 (14)	0.83	0.056	4.21	0.612 (16)	0.549	0.148	-1.43	− 0.542 (14)	0.596	− 0.14	5.24	0.774 (10)	0.457	0.233	− 0.21	− 0.039 (13)	0.97	− 0.01
Tower	1.47	-2.05(18)	0.055	− 0.47	0.733	− 0.75 (14)	0.46	− 0.194	1	− 0.834 (14)	0.418	− 0.215	**1.87**	**-2.114 (14)**	**0.053**	**− 0.546**	1.36	− 0.739 (10)	0.477	− 0.223	**2.86**	**-3.714 (13)**	**0.003**	**− 0.993**
CPT Commissions (t-scores)	6.57	2.037 (18)	0.057	0.467	**5.49**	**3.949 (14)**	**0.001**	**1.02**	-1.95	− 0.788 (15)	0.443	0.197	1.06	0.871 (14)	0.398	0.225	4.24	1.203 (9)	0.26	0.38	7.22	4.184 (13)	*p* < .001	1.118
WCST Category Completed	0.158	− 0.382 (18)	0.707	− 0.088	0.2	-1.382 (14)	0.189	− 0.357	− 0.13	0.522 (15)	0.609	0.131	0.13	0.619 (14)	0.546	0.16	0.9	-1.304 (9)	0.225	0.412	0.36	-1.00 (13)	0.336	0.267
WCST Perseverative Errors	2.947	1.627 (18)	0.121	0.373	2.133	1.896 (14)	0.079	0.49	− 0.63	− 0.41 (15)	0.688	0.102	1.4	1.408 (14)	0.181	0.364	5.2	1.389 (9)	0.198	0.439	3.79	1.864 (13)	0.085	0.498
**Attention**																								
DST	− 0.75	-1.617 (19)	0.122	− 0.362	0.40	0.823 (14)	0.424	0.213	**-1.0**	**-2.675 (16)**	**0.017**	**− 0.649**	-1.0	-1.511 (14)	0.153	− 0.39	− 0.666	− 0.0853(11)	0.412	− 0.246	− 0.857	-1.312(13)	0.212	− 0.351
DSST	-1.5	− 0.884 (19)	0.388	− 0.198	**-4.8**	**-2.636 (14)**	**0.02**	**− 0.681**	**-5.0**	**-2.901 (16)**	**0.010**	**− 0.704**	-2.066	-1.134 (14)	0.276	− 0.293	**-6.916**	**-3.06(11)**	**0.011**	**− 0.884**	**-6.7143**	**-3.337(13)**	**0.005**	**− 0.892**
CPT omission (t-scores)	10.25	1.954 (18)	0.066	0.448	-1.204	− 0.76 (14)	0.46	− 0.196	-6.17	− 0.489 (15)	0.632	− 0.122	**4.16**	**2.306 (14)**	**0.037**	**0.596**	-4.077	− 0.183(9)	0.859	− 0.058	2.905	2.118(13)	0.054	0.566
WCST Failure Maintain Set	− 0.263	− 0.925 (18)	0.367	− 0.212	− 0.066	− 0.138 (14)	0.892	− 0.036	− 0.375	-1.031 (15)	0.319	− 0.258	− 0.0667	− 0.122 (14)	0.905	− 0.031	**-1.00**	**-3.354 (9)**	**0.008**	**0.94**	− 0.2143	− 0.822 (13)	0.426	− 0.220

MC = mean change

*d* = cohens d

In sum, the patient group showed a significant long-term improvement in inhibition auditory attention, working memory, visual attention and processing speed, while controls did not demonstrate such improvement. The healthy controls showed long-term practice effects on several EF measures except in inhibition measured by CWIT-3. Both groups showed significant long-term improvement in visual attention, while only the patient group showed significantly more long-term inattention (See Table 4 for a summary of results).

**Table 4:** Summary of findings: differences in executive- and attentional functioning and changes in patients’ cognitive performance

	Pre ECT (T1)	Post ECT (T2)		6 months following ECT (T3)	
	Group differences (*p* < .05)	Group differences (*p* < .05)	Change in patient group from T1 (*p* < .05)	Group differences (*p* < .05)	Change in patient group from T1 (*p* < .05)
**Executive functions**	N Tests = 6/13	N Tests = 10/13	N Tests = 2/13	N Tests = 11/13	N Tests = 2/13
***Inhibition***					
CWIT-3 Inhibition	≠	≠	▲	≠	▲
CPT Commissions	≈	≈	≈	≠	≈
CWIT-3 Contrast	≠	≠	▲	0.068	▲
***Flexibility/ Shifting***					
CWIT-4 Inh/switch	0.069	≠	≈	≠	0.055
CWIT4 Contrast	≈	≠	≈	0.081	≈
Category Switching	≈	≠	0.055 (▼)	≠	≈
TMT-4 (TMT-B)	≠	≠	≈	≠	≈
TMT-4 Contrast	≠	≠	≈	≠	≈
WCST CC	≈	≈	≈	≈	≈
WCST PE	≈	≈	≈	≈	≈
***Verbal Fluency***					
Letter Fluency	0.090	≠	≈	≠	≈
Category Fluency	≠	≠	≈	≠	≈
***Planning ability***					
Tower Achievement	≠	≠	≈	≠	≈
**Attentional functioning**	N Tests = 3/4	N Tests = 2/4	N Tests = 2/4	N Tests = 2/4	N Tests = 2/4
***Auditory Attention Working Memory***					
Digit Span Test	≠	≈	▲	≈	≈
***Visual attention*** ***Processing speed***					
DSST	≠	≠	▲	≠	▲
***Inattention***					
CPT Omission	≠	0.059	0.066 (▲)	≠	≈
WCST FMS	≈	≈	≈	≈	▲

CC = Categories Completed, CPT = Continuous Performance Test-II, CWIT = Color-Word Interference Test, DSST = Digit Symbol Substitution Test, ECT = Electroconvulsive therapy, FMS = Failure to Maintain Set, PE = Perseverative Errors, WCST = Wisconsin Card Sorting Test, N Tests = number of tests in each domain significantly different between groups or improved /total number of tests, ≠ significant difference between groups, ▲ significant improvement, ≈ no significant difference between groups ≈ no significant change in test score

### The relation between symptoms and cognitive function

The bivariate correlation coefficients calculated for change scores in neuropsychological tests and change depressive symptoms did not reveal any significant results, except for inhibition measured by CWIT3 *ρ*(9) *=* 0.609, *p =* .047.

## Discussion

The present study aimed to investigate the short- and long-term effects of ECT on EF and attention. By exploiting the rapid antidepressant effect of ECT, changes in attentional and executive function in relation to trait/scar and state effects of depression were examined. The study found no indications of worsening general executive and attentional functions following ECT, as deficits apparent pre-treatment either persisted or improved following ECT. An improvement in specific aspects of EF and attention was evident in inhibition, visual attention, processing speed, auditory attention, and working memory. However, results varied between tests within aspects for several of the executive functions and in certain aspects of attention. While patients six months after ECT reached a level of functioning comparable to healthy controls in auditory attention and working memory, impairments were still evident in visual attention, processing speed, inattention, and executive functions. Thus, findings in the present study provide support for both perspectives, with a state-related cognitive functional improvement following a significant antidepressant effect of ECT, in addition to persistent cognitive impairments that may be attributed to trait- or scar-related effects of depression.

### Pre-existing cognitive deficits

Expected significant differences between groups at baseline were found for most but not all measures. This is in line with meta-studies of EF in MDD [20, 21]. Patients demonstrated significantly poorer ability to plan, as measured by the Tower Test. Patients showed deficits in one of the measures of inhibition (CWIT-3), while inhibition, as measured by CPT commissions, was not significantly poorer compared to controls, although this performance showed small to medium effect size. Also, for verbal fluency, patients performed significantly worse in the category fluency condition. There were impairments in one measure of the switching aspect or mental flexibility (TMT-4). Differences were not significant when switching was measured with CWIT-4, although differences approached significance and showed medium effect size with patients performing worse than controls. Patients also showed reduced capacity in all aspects of attention except in WCST failure to maintain set. This coincides with previous research pointing to impairments in various aspects of attention due to depression [33–35]. Regarding inattention, patients performed significantly poorer on CPT omissions compared to controls, while performance did not differ significantly on WCST failure to maintain set. One possible explanation for the differing results could be reaction time, and the latter might be less affected by the reduced processing speed. However, Stordal et al. [60] did find impairments in the WCST condition failure to maintain set al.so when adjusted for psychomotor speed.

Impairments, defined as significant group differences, were identified for all measured aspects of attention and EF. Finding impaired attentional and executive functioning in patients aligns with previous studies on the neurocognitive profile in patients with MDD [18–21]. However, given the significant differences between the groups in IQ and years of education, these differences might be overestimated by the current somewhat biased sample, which could be evident from the relatively large effects in the current study when compared to those reported in meta-analyses of cognition in the acute state of MDD [27]. However, patients with more treatment resistance (like the current sample) have been shown to have more deficits [20]. In addition, patients did not perform significantly differently from controls on four measures of EF, which could be explained by a small sample size and insufficient power to detect smaller effects consistently. Also, some tests of EF are likely less sensitive to the cognitive deficits experienced in MDD [25].

### Changes following electroconvulsive treatment

As hypothesized, EF and attentional capacity either improved or remained stable post-ECT. Results may be interpreted as supporting earlier findings, indicating that the domains of EF and attention are unharmed by ECT [9, 42, 47]. Results are similar, albeit somewhat larger than found in previous meta-studies of cognitive changes following ECT [15, 42]. Separating the domains of EF and attention, in addition to discerning the respective aspects of these domains, we were able to identify some nuances adding to previous results, suggesting that most aspects of these functions improve. This was also possible due to the comparison of potential practice effects in the patient and control group.

Patients did demonstrate an improvement in inhibition measured by CWIT3 from T1 to T3 that was not detected in controls. However, practice effects in inhibition were evident in the control group at the short-term follow-up and this might indicate that the control group had reached a ceiling effect at the long-term follow-up, or could be explained by lower statistical power to detect small changes. Contrary to a recent meta-analysis, the present study did not find a short-term decline in verbal fluency [15]. This could be explained by the low statistical power of the present study, but also the heterogeneity in time intervals for short-term measures and patient groups included in the meta-analysis [15]. Notably, our study found a possible short-term decline in category switching. However, this finding approaching statistical significance should be further investigated in larger studies with more statistical power. The control group demonstrated statistically significant improvements on several tests that were not found in patients. These results indicate that patients have lost practice effects, which could be either due to the depressive state, ECT, or an interaction of both. Alternatively, or in addition, the control group showed less dropout and thus, to a larger extent, had incremental practice effects in all participants that could also partly explain larger practice effects.

In sum, there was no decline in patients’ performance on executive measurements, but a significant long-term improvement in patients´ ability to inhibit. Compared to patients, the control participants demonstrated greater practice effects. This could indicate a missed practice effect for patients in measures of EF, or it could indicate a negative effect of treatment on performance. In this regard, the results do not rule out the possibility of iatrogenic effects of ECT on EF. However, the preexisting differences in most cognitive tests, in addition to the known effects of depression on neuroplasticity [61], suggest that depression history, at least partly, could explain the relatively poor practice effects in the depression group [18]. This could be especially true for the current patient group undergoing ECT considering this treatment often is administered for particularly complex and severe cases of MDD.

The patient group showed long-term improvement in processing speed and visual attention as expected. Results could thus indicate a positive effect of the symptom recovery following ECT on these aspects of attentional functioning. However, the control group also showed a long-term significant improvement in visual attention and processing speed, which may indicate that patients’ improvements may be the result of the practice effects discussed above. Results regarding processing speed support previous findings on how this aspect of attention is affected by the presence of depressive symptoms [29], and results could therefore support the state hypothesis. Also, a recent meta-analysis found processing speed improved following remission which also supports this perspective [17]. Still, group differences were present at all time points also indicating a trait or scar perspective on attention. Patients demonstrated a long-term improvement in auditory attention and working memory, where the latter is in line with previous findings [9, 44, 45, 47]. Both groups showed significantly less long-term inattention, indicating practice effects.

### Persisting deficits and their relations to symptoms

Patients were expected to demonstrate residual impairment in both EF and attention six months post-ECT [18, 22, 26, 28, 29, 37, 62]. As expected, patients performed significantly poorer compared to the control group on most EF variables at both the short- and long-term follow-up (T2 and T3). The results for attentional assessments were slightly more diverse. At the short-term follow-up, results indicated comparable results for auditory attention, working memory, and inattention, but not for processing speed and visual attention. Results also indicated comparable abilities for auditory attention and working memory at the long-term follow-up. Significant differences were found in visual attention, processing speed, and inattention at the six-month follow-up.

With some exceptions, results indicate stable differences in performance for EF, inattention, visual attention, and processing speed from pre- to post-treatment. Patients showed a stable tendency to score lower in these aspects in agreement with previous research showing deficits beyond the depressed episode [17, 18, 28, 29, 37]. Again it’s worth mentioning the significant differences in important demographic variables could have inflated these effects, as deficits are larger than those reported in a large meta-analysis of remitted depression [18]. However, given the severe depression experienced by the patient group, larger deficits would be expected. Thus, the present findings mostly support persisting deficits that were evident before ECT treatment. Such findings align with the trait- and scar hypothesis for depression. However, indications for a state effect were also identified through findings showing that patients reached a level of functioning comparable to healthy controls on post-treatment measures in certain aspects. Results could also be influenced by MADRS scores indicating considerable residual symptoms in the patient group at follow-up. Finally, since there were no tests of the cognitive functioning of the patients before their illness, the trait perspective may only be inferred, not confirmed by the current study design.

A relationship between improvement in mood symptoms and cognitive functions was expected. The present study only found such an association for inhibition measured by the CWIT. Finding that a change in inhibition score was related to the change in depressive symptoms adds to previous findings that indicate a pattern of trait-related effects of depression on inhibition [36] showing significant differences in inhibition between groups despite mood symptom load. The correlation between improved inhibition and mood identified in the present study aligns with the state hypothesis, predicting that inhibition improves as the depressive symptoms decrease. Alternatively, or in addition, improvements in inhibition could be related to the antidepressant effects of ECT. However, the limited statistical power of this study and the small sample showing these effects warrants careful interpretations, and there is a need to test this relation with a larger sample size. Also, the low variance in depressive scores might have an impact on the present findings as well as the potential practice effects related to findings of improvement in the healthy control group as well. Still, inspecting the long-term improvements in attention and psychomotor speed measures along with significant symptom improvement, might support a state perspective for these functions.

### Strengths, limitations and further directions

The present study has several strengths and limitations that are recommended to be considered in future research. The longitudinal design enabled monitoring of changes over time and exploring the cognitive nature of depression as well as both short- and long-term effects of ECT. However, future studies should also include longitudinal measurements beyond six months to further investigate long-term changes. The inclusion of a healthy control group has previously been a methodological shortcoming in ECT research [15], and is included in the present study to identify deficits present pre-treatment. By including a healthy control group, we also controlled for potential practice effects and were able to differentiate between impairments attributable to treatment from impairments related to depression per se.

Heterogeneity in the use of ECT in research challenges the ability to compare results and may have contributed to inconclusive or mixed findings regarding the cognitive side-effects of treatment. It is not uncommon that studies lack a differentiation between different ECT procedures [42, 47]. This represents a challenge when modern and older ECT practices are associated with different outcomes in cognitive function [15]. Consequently, standardization of ECT procedures should be pursued. Further, there is a need for standardizing test batteries used in ECT studies, as there is great variation in the application of neurocognitive tests. To account for this, a variety of established neuropsychological tests were administered in the present study, and such cognitive tests are strongly recommended to be included in routine ECT follow-up [15]. Also, our study indicates that some aspects of WCST are not sensitive to cognitive deficits in MDD.

A limitation of the present study is that it did not include any self-report measures, which may lead to an incomplete understanding of the extent of persistent or improved cognitive difficulties. Autobiographical memory loss is often of concern following ECT [16]. The discrepancy between subjective and objective outcomes after ECT [46] should be explored further and subjective measures included to provide a more comprehensive understanding of cognitive change.

Matching for demographic variables could be important in research on MDD [14, 63]. In the present study, both patients and healthy controls were comprehensively examined in terms of demographic parameters. Correspondingly, the control group had statistically significant higher education and IQ, which may have influenced cognition and thus the validity of the present results as discussed above. In future research, we suggest efforts are made to match demographic variables between patients and healthy controls, however, this is not always indicated, e.g. when the condition being studied influences these aspects [64]. The small sample might have resulted in overestimated effect sizes. In addition, there was no upper limit on the age of included participants, and even though the groups did not significantly differ on this variable, age could have influenced results. In conclusion, larger samples powered to perform multivariate analysis that control for covariates such as age and depressive symptoms could be important for future investigations of the effects of ECT on cognition.

The lack of a structured clinical interview at inclusion could be considered a limitation, but the inclusion of patients with both bipolar and unipolar depressive disorder gives the study a broad ecologically valid, and representative, sample. However, both groups consist of small samples, making subgroup comparisons difficult. In addition, the current study has a substantial amount of missing data. Late inclusion of patients and a high drop-out rate are the reason for this. Because of missing data and sample size, it is possible that some group differences or changes in attention and EF were undetected and that findings were influenced by attrition effects. In addition, the statistical approach utilizes several statistical comparisons enhancing the risk for type-I errors. However, given the novel nature of the study, type-II errors represent a risk of underestimating difficulties in this vulnerable group. Results should be interpreted accordingly. Further examination is needed to clarify how ECT and depression affect cognitive functions in patients with severe and treatment-resistant depression, in large, matched samples, with long follow-up time and tests assessing several domains of cognition, and novel results from this study should be considered preliminary until replicated in an independent sample. To best address the potential iatrogenic effects of ECT, patients with depression matched on clinical and demographic variables could be compared on ECT if one group underwent ECT, and one did not.

### Clinical implications

Results from this study are useful for both practitioners and patients. Findings may contribute to providing patients with more nuanced information regarding potential side effects of ECT treatment, in addition to awareness about impairments that may be attributed to the depression itself, and that the deficits reported by patients may be influenced secondary to reduced EF and attention caused by ECT. Even after treatment, our results show lasting cognitive difficulties when compared to a healthy group. Such knowledge may be used to normalize patients’ experience of cognitive impairments. Also, findings suggest that cognitive remediation interventions could be useful in this group [65].

It is important to acknowledge that our results are based on group-level analyses and hence cannot be used to refute the fact that a few patients may experience adverse effects following ECT [66]. Also, it is known that autobiographical memory consistency is reduced following ECT [67], but this was not investigated in the present study.

More detailed knowledge about the side-effects of ECT as provided in this study may strengthen clinicians’ and patients´ ability to make informed decisions about ECT treatment and predict risks and benefits associated with treatment. Importantly, by finding mostly positive and neutral effects of ECT on tests of EF and attention, this study may contribute to reducing the stigma associated with the treatment. Reducing stigma and clarifying side-effects could be a key factor to minimize underutilization of a treatment option that besides being lifesaving has the potential to improve patients’ functioning and quality of life.

## Conclusions

Overall, this study did not find indications of worsening of performance after ECT on either aspects of attention or executive functions in patients with depression. However, the control group showed improved performance in some aspects and this might indicate lost practice effects for the patient group. Patients demonstrated both short- and long-term improvement in several aspects of cognitive function, alluding to a possible state-related improvement following symptom relief, still with a significant difference in performance compared to the control group. More specifically, patients showed significant improvement in auditory attention, working memory, visual attention, processing speed, and inhibition from pre- to post-measures. However, group differences identified both pre- and post-ECT indicated persistent impairments in EF and some aspects of attention (visual attention, processing speed and inattention) that are interpreted as supporting a trait- or scar effect of depression on cognitive function. In conclusion, it may be inferred that the cognitive profile following ECT is differential, with some aspects of cognitive functions improving along with symptom reduction, while others remain impaired related to the enduring effects of depression or inherent vulnerability traits.

## Data Availability

Anonymized data will be made available per inquiry in accordance with Norwegian data protection laws.

## Abbreviations

ECT: Electroconvulsive treatment
MDD: Major depressive disorder
MADRS: Montgomery Aasberg Depression Rating Scale
IQ: Intelligence quotient
D-KEFS: Delis-Kaplan Executive Function System
WCST: Wisconsin Card Sorting Test
CPT: Continuous Performance Test
CWIT: Color-Word Interference Test
TMT: Trail Making Test
DSST: Digit Symbol Substitution Test
DST: Digit Span Test

## References

[1] Scott KM, De Jonge P, Stein DJ, Kessler RC. redaktører. Mental disorders around the World: facts and figures from the World Mental Health Surveys [Internett]. 1. Utg. Cambridge University Press; 2018. [sitert 19. oktober 2024]. Tilgjengelig på. https://www.cambridge.org/core/product/identifier/9781316336168/type/book.

[2] Moffitt TE, Caspi A, Taylor A, Kokaua J, Milne BJ, Polanczyk G. Mfl. How common are common mental disorders? Evidence that lifetime prevalence rates are doubled by prospective *versus* retrospective ascertainment. Psychol Med juni. 2010;40(6):899–909. 10.1017/S0033291709991036 PMC3572710 19719899

[3] Otte C, Gold SM, Penninx BW, Pariante CM, Etkin A, Fava M. Mfl. Major depressive disorder. Nat Reviews Disease Primers 15 September. 2016;2(1):16065. 10.1038/nrdp.2016.65 27629598

[4] Lépine JP, Briley M. The increasing burden of depression. Neuropsychiatr Dis Treat. 2011;7(Suppl 1):3–7. 21750622 10.2147/NDT.S19617 PMC3131101

[5] Miller S, Dell’Osso B, Ketter TA. The prevalence and burden of bipolar depression. J Affect Disord Desember. 2014;169(Suppl 1):S3–11. 10.1016/S0165-0327(14)70003-5 25533912

[6] McIntyre RS, Alsuwaidan M, Baune BT, Berk M, Demyttenaere K, Goldberg JF. Mfl. Treatment-resistant depression: definition, prevalence, detection, management, and investigational interventions. World Psychiatry Oktober. 2023;22(3):394–412. 10.1002/wps.21120 PMC10503923 37713549

[7] Kho KH, Van Vreeswijk MF, Simpson S, Zwinderman AH. september. A Meta-analysis of Electroconvulsive Therapy Efficacy in Depression: the Journal of ECT. 2003;19(3):139–47. 10.1097/00124509-200309000-00005 12972983

[8] Pagnin D, De Queiroz V, Pini S, Cassano GB. Efficacy of ECT in Depression: A Meta-Analytic Review: The Journal of ECT. mars. 2004;20(1):13–20. 10.1097/00124509-200403000-00004 15087991

[9] Trifu S, Sevcenco A, Stănescu M, Drăgoi A, Cristea M. Efficacy of electroconvulsive therapy as a potential first–choice treatment in treatment–resistant depression (review). Exp Ther Med. september 2021;9(5):1281. 10.3892/etm.2021.10716 PMC8461517 34630636

[10] Efficacy. Safety of electroconvulsive therapy in depressive disorders: a systematic review and meta-analysis. Lancet mars. 2003;361(9360):799–808. 10.1016/S0140-6736(03)12705-5 12642045

[11] Ziegelmayer C, Hajak G, Bauer A, Held M, Rupprecht R, Trapp W. Cognitive performance under Electroconvulsive Therapy (ECT) in ECT-Naive treatment-resistant patients with Major Depressive Disorder. J ECT Juni. 2017;33(2):104–10. 10.1097/YCT.0000000000000385 28169947

[12] Li M, Yao X, Sun L, Zhao L, Xu W, Zhao H. Mfl. Effects of Electroconvulsive Therapy on Depression and its potential mechanism. Front Psychol 20 Februar. 2020;11:80. 10.3389/fpsyg.2020.00080 PMC7044268 32153449

[13] Liang CS, Chung CH, Tsai CK, Chien WC. In-hospital mortality among electroconvulsive therapy recipients: a 17-year nationwide population-based retrospective study. Eur Psychiatr Mai. 2017;42:29–35. 10.1016/j.eurpsy.2016.12.005 28199870

[14] Aoki Y, Yamaguchi S, Ando S, Sasaki N, Bernick PJ, Akiyama T. The experience of electroconvulsive therapy and its impact on associated stigma: a meta-analysis. Int J Soc Psychiatry Desember. 2016;62(8):708–18. 10.1177/0020764016675379 27798050

[15] Landry M, Moreno A, Patry S, Potvin S, Lemasson M. Current practices of Electroconvulsive Therapy in Mental disorders: a systematic review and Meta-analysis of short and long-term Cognitive effects. J ECT Juni. 2021;37(2):119–27. 10.1097/YCT.0000000000000723 33009218

[16] Porter RJ, Baune BT, Morris G, Hamilton A, Bassett D, Boyce P. Mfl. Cognitive side-effects of electroconvulsive therapy: what are they, how to monitor them and what to tell patients. BJPsych open mai. 2020;6(3):e40. 10.1192/bjo.2020.17 PMC7191622 32301408

[17] Ahern E, White J, Slattery E. Change in cognitive function over the course of major depressive disorder: a systematic review and Meta-analysis. Neuropsychol Rev [Internett]. 5. Februar 2024 [sitert 2. september 2024]; Tilgjengelig på: https://link.springer.com/10.1007/s11065-023-09629-9 10.1007/s11065-023-09629-9 38315296

[18] Semkovska M, Quinlivan L, O’Grady T, Johnson R, Collins A, O’Connor J. Mfl. Cognitive function following a major depressive episode: a systematic review and meta-analysis. Lancet Psychiatry Oktober. 2019;6(10):851–61. 10.1016/S2215-0366(19)30291-3 31422920

[19] Nuño L, Gómez-Benito J, Carmona VR, Pino O. A systematic review of executive function and information Processing Speed in Major Depression Disorder. Brain Sci 22 Januar. 2021;11(2):147. 10.3390/brainsci11020147 PMC7912411 33499360

[20] Rhee TG, Shim SR, Manning KJ, Tennen HA, Kaster TS, d’Andrea G. Mfl. Neuropsychological assessments of cognitive impairment in major depressive disorder: a systematic review and Meta-analysis with Meta-regression. Psychother Psychosom. 2024;93(1):8–23. 38272009 10.1159/000535665 PMC10880806

[21] Snyder HR. Major depressive disorder is associated with broad impairments on neuropsychological measures of executive function: a meta-analysis and review. Psychol Bull. 2013;139(1):81–132. 22642228 10.1037/a0028727 PMC3436964

[22] Gudayol-Ferré E, Duarte-Rosas P, Peró-Cebollero M, Guàrdia-Olmos J, THE EFFECT OF SECOND-GENERATION ANTIDEPRESSANT, TREATMENT ON THE EXECUTIVE FUNCTIONS OF PATIENTS WITH MAJOR DEPRESSIVE DISORDER. A META-ANALYSIS STUDY WITH STRUCTURAL EQUATION MODELS. Psychiatry Res 1 Februar. 2021;296:113690. 10.1016/j.psychres.2020.113690 33387749

[23] Allott K, Fisher CA, Amminger GP, Goodall J, Hetrick S. Characterizing neurocognitive impairment in young people with major depression: state, trait, or scar? Brain Behav Oktober. 2016;6(10):e00527. 10.1002/brb3.527 PMC5064339 27781141

[24] Luo R, Fan N, Dou Y, Wang Y, Wang M, Yang X. Mfl. Relationship between cognitive function and functional outcomes in remitted major depression. BMC Psychiatry 24 April. 2024;24(1):311. 10.1186/s12888-024-05675-6 PMC11040809 38658936

[25] Snyder HR, Miyake A, Hankin BL. Advancing understanding of executive function impairments and psychopathology: bridging the gap between clinical and cognitive approaches. Front Psychol [Internett]. 2015. 10.3389/fpsyg.2015.00328. https://www.frontiersin.org/journals/psychology/articles/. 6. Tilgjengelig på. 25859234 10.3389/fpsyg.2015.00328 PMC4374537

[26] Årdal G, Hammar Å. Is impairment in cognitive inhibition in the acute phase of major depression irreversible? Results from a 10-year follow‐up study. Psychol Psychother Juni. 2011;84(2):141–50. 10.1348/147608310X502328 22903853

[27] Parkinson WL, Rehman Y, Rathbone M, Upadhye S. Performances on individual neurocognitive tests by people experiencing a current major depression episode: a systematic review and meta-analysis. J Affect Disorders 1 November. 2020;276:249–59. 10.1016/j.jad.2020.07.036 32697706

[28] Douglas KM, Porter RJ. Longitudinal Assessment of Neuropsychological function in Major Depression. Aust N Z J Psychiatry Desember. 2009;43(12):1105–17. 10.3109/00048670903279887 20001409

[29] Ronold EH, Schmid MT, Oedegaard KJ, Hammar Å. A longitudinal 5-Year Follow-Up study of cognitive function after First Episode Major Depressive Disorder: exploring State, Scar and Trait effects. Front Psychiatry 7 Desember. 2020;11:575867. 10.3389/fpsyt.2020.575867 PMC7750430 33364989

[30] Rosario Rueda M, Pozuelos P, Cómbita JM L, Dept. of Experimental Psychology, Center for Research on Mind, Brain, and, Behavior. (CIMCYC), Universidad de Granada, Spain. Cognitive Neuroscience of Attention From brain mechanisms to individual differences in efficiency. AIMS Neuroscience. 2015;2(4):183–202.

[31] Lindsay GW. Attention in psychology, Neuroscience, and machine learning. Front Comput Neurosci. april 2020;16:14:29. 10.3389/fncom.2020.00029 PMC7177153 32372937

[32] Treviño M, Zhu X, Lu YY, Scheuer LS, Passell E, Huang GC. Mfl. How do we measure attention? Using factor analysis to establish construct validity of neuropsychological tests. Cogn Res Desember. 2021;6(1):51. 10.1186/s41235-021-00313-1 PMC8298746 34292418

[33] Wang X, Zhou H, Zhu X. Attention deficits in adults with major depressive disorder: a systematic review and meta-analysis. Asian J Psychiatry Oktober. 2020;53:102359. 10.1016/j.ajp.2020.102359 32891927

[34] Kriesche D, Woll CFJ, Tschentscher N, Engel RR, Karch S. Neurocognitive deficits in depression: a systematic review of cognitive impairment in the acute and remitted state. Eur Arch Psychiatry Clin Neurosci August. 2023;273(5):1105–28. 10.1007/s00406-022-01479-5 PMC10359405 36048295

[35] Little B, Anwyll M, Norsworthy L, Corbett L, Schultz-Froggatt M, Gallagher P. Processing speed and sustained attention in bipolar disorder and major depressive disorder: a systematic review and meta‐analysis. Bipolar Disorders mars. 2024;26(2):109–28. 10.1111/bdi.13396 37973384

[36] Hammar Å, Ronold EH, Rekkedal GÅ. Cognitive impairment and neurocognitive profiles in Major Depression—A clinical perspective. Front Psychiatry [Internett]. 2022. 10.3389/fpsyt.2022.764374. https://www.frontiersin.org/journals/psychiatry/articles/. 13. Tilgjengelig på. 35345877 10.3389/fpsyt.2022.764374 PMC8957205

[37] Gudayol-Ferré E, Duarte-Rosas P, Peró-Cebollero M, Guàrdia-Olmos J. The effect of second-generation antidepressant treatment on the attention and mental processing speed of patients with major depressive disorder: a meta-analysis study with structural equation models. Psychiatry Res. august 2022;1:314:114662. 10.1016/j.psychres.2022.114662 35689972

[38] Bernhardt M, Klauke S, Schröder A. Longitudinal course of cognitive function across treatment in patients with MDD: a meta-analysis. J Affect Disorders April. 2019;249:52–62. 10.1016/j.jad.2019.02.021 30753954

[39] Kessler U, Schoeyen HK, Andreassen OA, Eide GE, Hammar Å, Malt UF. Mfl. Neurocognitive profiles in treatment-resistant bipolar I and bipolar II disorder depression. BMC Psychiatry Desember. 2013;13(1):105. 10.1186/1471-244X-13-105 PMC3637095 23557429

[40] Malekizadeh H, Saed O, Rashtbari A, Sajjadi M, Ahmadi D, Ronold EH. Deficits in specific executive functions manifest by severity in major depressive disorder: a comparison of antidepressant naïve inpatient, outpatient, subclinical, and healthy control groups. Front Psychiatry. oktober 2023;3:14:1225062. 10.3389/fpsyt.2023.1225062 PMC10580982 37854445

[41] Ronold E, Schmid MT, Hammar Å. Risk factors and cognitive deficits in First Episode Major Depression: a five-year longitudinal study of explorative subgroups. Biol Psychiatry Mai. 2021;89(9):S131.

[42] Semkovska M, McLoughlin DM. Objective Cognitive Performance Associated with Electroconvulsive Therapy for Depression: a systematic review and Meta-analysis. Biol Psychiatry September. 2010;68(6):568–77. 10.1016/j.biopsych.2010.06.009 20673880

[43] Kessler U, Schoeyen HK, Andreassen OA, Eide GE, Malt UF, Oedegaard KJ. Mfl. The Effect of Electroconvulsive Therapy on neurocognitive function in treatment-resistant bipolar disorder depression. J Clin Psychiatry 24 November. 2014;75(11):e1306–13. 10.4088/JCP.13m08960 25470096

[44] Mohn C, Rund BR. Neurocognitive profile in major depressive disorders: relationship to symptom level and subjective memory complaints. BMC Psychiatry 19 April. 2016;16:108. 10.1186/s12888-016-0815-8 PMC4837617 27095362

[45] Mohn C, Rund BR. Maintained Improvement of Neurocognitive Function in Major Depressive Disorders 6 Months after ECT. Front Psychiatry [Internett]. 19. desember 2016 [sitert 2. september 2024];7. Tilgjengelig på: http://journal.frontiersin.org/article/10.3389/fpsyt.2016.00200/full 10.3389/fpsyt.2016.00200 PMC5165021 28066273

[46] Xu Sxian, Xie X, hui, Yao L, Chen L, chang, Wan Q, Chen Z. hua, mfl. Trajectories of Efficacy and Cognitive Function During Electroconvulsive Therapy Course in Young Adults with Treatment-Resistant Depression. NDT. januar 2023;Volume 19:267–81. 10.2147/NDT.S394155 PMC9893845 36744206

[47] Vasavada MM, Leaver AM, Njau S, Joshi SH, Ercoli L, Hellemann G. Mfl. Short- and long-term cognitive outcomes in patients with Major Depression treated with Electroconvulsive Therapy. J ECT Desember. 2017;33(4):278–85. 10.1097/YCT.0000000000000426 PMC5705261 28617690

[48] Blomberg MO, Semkovska M, Kessler U, Erchinger VJ, Oedegaard KJ, Oltedal L. mfl. A Longitudinal Comparison Between Depressed Patients Receiving Electroconvulsive Therapy and Healthy Controls on Specific Memory Functions. Prim Care Companion CNS Disord [Internett]. 14. mai 2020 [sitert 2. september 2024];22(3). Tilgjengelig på: https://www.psychiatrist.com/pcc/memory-and-ect 10.4088/PCC.19m02547 32408397

[49] Porter RJ, Robinson LJ, Malhi GS, Gallagher P. The neurocognitive profile of mood disorders – a review of the evidence and methodological issues. Bipolar Disorders Desember. 2015;17(S2):21–40. 10.1111/bdi.12342 26688288

[50] Oltedal L, Kessler U, Ersland L, Grüner R, Andreassen OA, Haavik J. Mfl. Effects of ECT in treatment of depression: study protocol for a prospective neuroradiological study of acute and longitudinal effects on brain structure and function. BMC Psychiatry Desember. 2015;15(1):94. 10.1186/s12888-015-0477-y PMC4422607 25927716

[51] Helsedirektoratet. Nasjonal faglig retningslinje om bruk av elektrokonvulsiv behandling - ECT [Internett]. Tilgjengelig på: https://www.helsedirektoratet.no/retningslinjer/elektrokonvulsiv-behandling-ect/Elektrokonvulsiv%20behandling%20(ECT)%20%E2%80%93%20Nasjonal%20faglig%20retningslinje.pdf/_/attachment/inline/a0817fd2-8503-4ee6-93e3-9e6bd5fb14f7:e3b568bdee57d0a6124f3f34556e2d02aa36cf6c/Elektrokonvulsiv%20behandling%20(ECT)%20%E2%80%93%20Nasjonal%20faglig%20retningslinje.pdf

[52] Montgomery SA, Asberg M. A new depression scale designed to be sensitive to change. Br J Psychiatry April. 1979;134:382–9. 10.1192/bjp.134.4.382 444788

[53] Wechsler D. Wechsler Adult Intelligence Scale–Third edition. San Antonio, TX: Psychological Corporation; 1997.

[54] Delis DC, Kapland E, Kramer JH. Examiner’s manual of the Delis-Kaplan Executive Functioning System. The Psychological Corporation.; 2001.

[55] Heaton RK, Chelune GJ, Talley JL et al. Wisconsin card sorting test manual – revised and expanded. Psychological Assessment Resources.

[56] Conners CK. Conners’ continuous performance test (CPTII). Technical guide and software manual. Multi Health Systems.; 2002.

[57] Lenhard W, Lenhard A. Computation of Effect Sizes [Internett]. Unpublished; 2017 [sitert 2. september 2024]. Tilgjengelig på: http://rgdoi.net/10.13140/RG.2.2.17823.92329

[58] Ronold EH, Joormann J, Hammar Å. Computerized Working Memory Training in Remission from Major Depressive Disorder: effects on emotional Working Memory, Processing Speed, executive functions, and associations with symptoms. Front Behav Neurosci. 2022;16:887596. 35832292 10.3389/fnbeh.2022.887596 PMC9272008

[59] Cohen J. Statistical Power Analysis for the behavioral sciences. I United Kingdom: Taylor & Francis Group; 1988.

[60] Stordal KI, Lundervold AJ, Egeland J, Mykletun A, Asbjørnsen A. Landrø NI, mfl. Impairment across executive functions in recurrent major depression. Nordic J Psychiatry Januar. 2004;58(1):41–7. 10.1080/08039480310000789 14985153

[61] Sanacora G, Yan Z, Popoli M. The stressed synapse 2.0: pathophysiological mechanisms in stress-related neuropsychiatric disorders. Nat Rev Neurosci Februar. 2022;23(2):86–103. 10.1038/s41583-021-00540-x 34893785

[62] Boeker H, Schulze J, Richter A, Nikisch G, Schuepbach D, Grimm S. Sustained cognitive impairments after clinical recovery of severe depression. J Nerv Mental Disease September. 2012;200(9):773–6. 10.1097/NMD.0b013e318266ba14 22922242

[63] Frid LM, Kessler U, Ousdal OT, Hammar Å, Haavik J, Riemer F. Mfl. Neurobiological mechanisms of ECT and TMS treatment in depression: study protocol of a multimodal magnetic resonance investigation. BMC Psychiatry 30 Oktober. 2023;23(1):791. 10.1186/s12888-023-05239-0 PMC10617235 37904091

[64] Dennis M, Francis DJ, Cirino PT, Schachar R, Barnes MA, Fletcher JM. Why IQ is not a covariate in cognitive studies of neurodevelopmental disorders. J Int Neuropsychol Soc mai. 2009;15(3):331–43. 10.1017/S1355617709090481 PMC3075072 19402919

[65] Van De Velde N, Kappen M, Koster EHW, Hoorelbeke K, Tandt H, Verslype P. Mfl. Cognitive remediation following electroconvulsive therapy in patients with treatment resistant depression: randomized controlled trial of an intervention for relapse prevention – study protocol. BMC Psychiatry Desember. 2020;20(1):453. 10.1186/s12888-020-02856-x PMC7493867 32938410

[66] Sellevåg K, Bartz-Johannessen CA, Oedegaard KJ, Nordenskjöld A, Mohn C. Bjørke JS, mfl. Unmasking patient diversity: exploring Cognitive and Antidepressive effects of Electroconvulsive Therapy. Eur Psychiatr 12 Januar 2024;1–34. 10.1192/j.eurpsy.2024.1 PMC10964271 38214065

[67] Semkovska M, Knittle H, Leahy J, Rasmussen JR. Subjective cognitive complaints and subjective cognition following electroconvulsive therapy for depression: a systematic review and meta-analysis. Aust N Z J Psychiatry Januar. 2023;57(1):21–33. 10.1177/00048674221089231 35362328

